# Meaningful engagement through critical reflexivity: Engaging people with lived experience in continuing mental health professional development

**DOI:** 10.1111/hex.13798

**Published:** 2023-06-26

**Authors:** Holly Harris, Chantalle Clarkin, Jordana Rovet, Allison Crawford, Andrew Johnson, Anne Kirvan, Sam Gruszecki, Stephanie Wang, Sophie Soklaridis

**Affiliations:** ^1^ Department of Education Centre for Addiction and Mental Health Toronto Ontario Canada; ^2^ Talk Suicide Canada Centre for Addiction and Mental Health Toronto Ontario Canada; ^3^ Bloomberg Faculty of Nursing University of Toronto Toronto Ontario Canada; ^4^ Virtual Mental Health and Outreach Centre for Addiction and Mental Health Toronto Ontario Canada; ^5^ Department of Psychiatry, Temerty Faculty of Medicine University of Toronto Toronto Ontario USA; ^6^ Health Out Loud Toronto Ontario Canada

**Keywords:** continuing professional development, critical reflexivity, engagement, lived experience, mental health

## Abstract

Engaging people with lived experience of mental health system encounters in the design and actualization of continuing professional development initiatives for mental health professionals can have transformative systemic impacts. Yet, despite evidence that involving people with lived experience benefits mental health professional education, far less focus has been placed on how to engage people with lived experience in continuing professional development initiatives. Tensions persist regarding the role of lived experience perspectives in continuing professional development, as well as how to establish people with lived experience as partners, educators and leaders in a thoughtful way. We propose that meaningful and equitable partnerships with people with lived experience can be realized by engaging in critical reflexivity and by systematically challenging assumptions. This paper explores three topics: (1) the current state of engagement with people with lived experience in continuing professional development initiatives; (2) barriers to meaningful engagement and (3) recommendations for using critical reflexivity to support the involvement and leadership of people with lived experience in continuing professional development for mental health professionals.

**Patient or Public Involvement:**

This viewpoint manuscript was co‐designed and co‐written by people with diverse lived and learned experiences. Each author's professional roles involve meaningfully and equitably partnering with and centring the perspectives of those with lived experience of mental health system encounters. In addition, approximately half of the authorship team identifies as having lived experience of accessing the psychiatric system and/or supporting family members who are navigating challenges related to mental health. These lived and learned experiences informed the conception and writing of this article.

## 1 INTRODUCTION

Engaging people with lived experience (PWLE) of mental health system encounters in the development and leadership of continuing professional development (CPD) initiatives for mental health professionals can have transformative systemic impacts.^1^ This involvement can infuse CPD initiatives with real‐world insights on health, health systems navigation and approaches that reflect compassionate, humanistic and recovery‐oriented care.^2^, ^3^ Recovery‐oriented care aligns with the Institute of Medicine's first core competency: to ‘identify, respect and care about patients' differences, values and expressed needs’.^4^, ^5^

Despite evidence that engaging PWLE benefits mental health professional education, far less emphasis has been placed on how to meaningfully engage PWLE in CPD initiatives.^3^ Tensions persist regarding the role of lived experience perspectives in CPD, as well as how to thoughtfully establish PWLE as partners, educators and leaders in CPD. We propose that meaningful and equitable partnerships with PWLE can be achieved through critical reflexivity and by systematically challenging assumptions.^6^ Critical reflexivity promotes exploration of knowledge generation through meaningful engagement, including different types of knowers and encourages both learners and educators to question how power structures influence the way that knowledge is generated.^6^ This paper explores three topics: (1) the current state of PWLE engagement in CPD initiatives; (2) barriers to meaningful engagement and (3) practical recommendations for supporting the involvement and leadership of PWLE in CPD for mental health professionals through critical reflexivity.

## 2 REFLEXIVITY AND POSITIONALITY

This paper was inspired by a workshop on the meaningful involvement of PWLE in CPD for mental health professionals, which was delivered by seven of this article's authors. As an interdisciplinary group that includes diverse learned and lived expertise, we continually challenge ourselves to engage in ongoing learning and advocacy, with the goal of moving toward more meaningful and ethical engagement of PWLE in our work. We encourage readers to do the same, regardless of where they are in this process. Given that our intersectional experiences have shaped how we conceptualize, approach and write on this topic, we include positionality statements for each author (see Table 1). We invite readers to consider the perspectives and positions we write from as well as the voices that have been left out.

**Table 1:** Authors' positionality statements.

Name	Positionality statement
Holly Harris	I acknowledge the intersectional privilege/oppression that I experience on account of my identity. I am a white, middle‐class, cisgender female with master's‐level education. I identify as someone who is neurodivergent and a consumer/survivor of the psychiatric system. I am employed by a tertiary mental healthcare facility as a research coordinator and have been working in community‐engaged research and programming for the past 5 years. I leverage my lived experiences as a source of strength, resilience and expertise to highlight the voices of those who have been historically silenced. I acknowledge that my lived, academic and professional experiences influence the value I place on specific ideas and my interpretation of data.
Chantalle Clarkin	Aspects of my identity and social location confer power, access and privilege. I am a white, queer, cisgender female living with a stable mental illness. I studied nursing in a small CÉGEP programme and have 22 years of experience as a registered nurse, working in a variety of hospital, community and clinical research contexts. I was the first in my family to complete a university degree, and my educational journey includes a master's degree in nursing and a doctorate in education. I am employed full‐time as a staff scientist in a large mental health organization, where I conduct community‐engaged research that is co‐designed with people with lived experience from start to finish. My personal and professional experiences, identities and social location shape how I come to understand myself and the world, and influence my research, scholarship and teaching practices. I believe that an authentic partnership with PWLE is key to disrupting power structures that maintain divisive and exclusionary hierarchies in health care, research and academia.
Jordana Rovet	I acknowledge that my lens for engaging with this paper has been shaped by my intersectional privileges, oppression, lived experiences and professional background. I am a white, cisgender female and a registered social worker with a master's degree. I have spent the last 10 years working alongside people with lived experience of mental health, substance use and addiction challenges, and I am acutely aware of the social and political context in which this work is embedded. I recognize the importance of actively reflecting on the tensions that I hold due to various aspects of my positionality and I am committed to engaging in a process of learning and unlearning.
Allison Crawford	I am a psychiatrist and scientist working in an academic health sciences centre. My social location is as an economically and socially advantaged, white, straight, cisgender female who is the first generation of my family to attend university. While I do not usually occupy the role of lived experience, I value the importance of critical reflexivity in my clinical and research work in mental health, particularly given the personal and familial engagement I have had with the medical and mental health system. I have benefited immensely from the interprofessional colleagues I work with, including people with lived and living experience. Throughout my work, I have often engaged with rich, diverse and equity‐seeking communities. I strive to critically reflect upon what those partnerships mean and to interrogate the role of my power and positionality in those engagements.
Andrew Johnson	I am a writer, editor, publisher and educator who works in the mental health space. For the past 25 years, I have worked in a large academic health science centre in a large Canadian city. Through progressively gaining experience and responsibility, I have reflected, and continue to reflect, on my ongoing privilege as a white, straight, middle‐aged man, and how that social location affects my commitments to deeply and authentically promoting inclusion, equity and diversity in all aspects of my professional life. To that end, the current phase of my career has given me an opportunity to lead the development of programmes that centre the voices of people with lived and living experiences of substance use, addiction and/or mental health challenges. To do so, I see my role as listening, sharing power and allowing others to step forward by me stepping back.
Anne Kirvan	I acknowledge that my perspectives and beliefs are shaped by my social location, as well as by my personal and professional experiences. I identify as a white cisgender female. I have a master's degree in social work, and I am employed as a clinical services consultant at a mental health and addiction hospital in a large Canadian city. I am also a PhD candidate in social work. I recognize the power and privilege associated with being an educator and researcher, and seek to use my positional power to collaboratively create spaces to meaningfully engage and partner with PWLE. I intend to continually learn from the perspectives and expertise that PWLE brings to this work, and to integrate that learning into my practice.
Sam Gruszecki	I identify as a white cisgender middle‐aged male. I work as a coordinator for a recovery college at an organization that employs many of the people involved with this paper. I had collegial and community‐based experience with most of them before starting this work. I am the child of an immigrant and lack postsecondary education. Some of my lived experience includes navigating anti‐Semitism, neurodivergence, multiple diagnoses and services and poverty. I have been involved in recovery college work, funded through major hospitals, as a peer support specialist, lead peer and coordinator since 2014. My experiences in research are relatively limited and I continue to learn along the way.
Stephanie Wang	As I engage throughout the development of this paper, I strive to identify, critique and consider the positionality from which I contribute. I am a managing director of a community‐based charity and also have other roles, including being part of a recovery college and CPD initiatives at a mental health hospital in a large Canadian city. I hope to acknowledge and reflexively contemplate the different forms of power, privileges and oppression that may be associated with the positions in which I am situated. This includes how I frequently partner with PWLE in educational, community and research contexts as someone who has my own experiences with mental health and identifies as a cisgender female from a multicultural background. My intent is to be open‐minded, learn and promote equity in health systems.
Sophie Soklaridis	I am the daughter of Greek parents who immigrated to Canada. I grew up in Lourdes, Newfoundland and Scarborough, ON, Canada. I hold assumptions and perspectives that are shaped by how I see/experience the world and how the world sees/experiences me. I am employed as a senior scientist and currently work across several academic medical institutions in Canada and Ethiopia. I recognize that the academic institutions I work in in Canada are privileged sites of North American knowledge production that have historically marginalized paradigms outside of a traditional biomedical model. My intent is to use my positional power to amplify the voices of colleagues, service users and family members as valued partners in the research process.

Abbreviations: CPD, continuing professional development; PWLE, people with lived experience.

## 3 CURRENT STATE OF PWLE INVOLVEMENT IN CPD

There is growing momentum to engage PWLE in the development and delivery of CPD. Power‐sharing is central to meaningfully and inclusively involving this population in CPD, whereby PWLE has the power to decide if, when and how they engage in these initiatives. However, most CPD initiatives for mental health professionals are still developed and delivered without the truly inclusive involvement of PWLE.^4^ Often, structure and support for engaging these voices are lacking, meaning that when opportunities for co‐production do arise, lived experience knowledge is marginalized.^7^ Rather than being recognized as equal partners, PWLEs are commonly engaged on an ad hoc basis and for a narrow aim (e.g., one‐off lectures), which does not move beyond the role of consultation.^7^, ^8^

Inviting people to share their stories is the most common form of engaging PWLE in CPD for mental health professionals.^7^ Storytelling can be a source of pride for PWLE and can shed light on important issues through sharing real‐world examples.^2^ However, stories are usually framed to complement a predetermined curriculum.^9^ When course leaders ask PWLE to frame their stories to fit established curricula, it can give the appearance of demonstrating support for the knowledge of PWLE by virtue of inclusion.^9^ However, establishing curriculum objectives without the meaningful involvement of PWLE is nothing more than superficial inclusion, which can reinforce power differentials between mental health professionals and the people they serve. To equitably partner with PWLE in CPD, we must question the current state of affairs and rethink how to involve PWLE in decision‐making.^7^, ^10^

## 4 BARRIERS TO INCLUSION OF PWLE IN CPD

There are social and structural barriers to the meaningful and inclusive involvement of PWLE in CPD. For example, most educational initiatives are designed to have one or two faculty co‐leads who lead with privileged forms of expertise (i.e., professional and/or academic). Often, there are no mechanisms for leaders of CPD initiatives to work with PWLE to define the scope for participation, thus reinforcing power hierarchies and barriers to equitable partnerships. A larger social issue to consider is the volunteer, underemployed and underpaid arrangements that constitute most roles for PWLE in CPD.^11^ Power dynamics are further perpetuated when those who are situated as leaders or administrators are afforded more stable employment opportunities as part of their professional roles, while PWLEs are constrained to precarious employment (e.g., contractual, part‐time, unpaid, lacking legal protection).^8^, ^12^, ^13^

Another barrier to equitable partnerships is the notion of professional acceptability. For instance, a study on the experiences of PWLE engaged in CPD found that programme leaders were more likely to offer opportunities to PWLE whom they deemed articulate and who had higher levels of education or existing relationships with clinicians.^14^ This suggests that PWLE who are considered ‘professionally acceptable’ are more likely to be involved in CPD. PWLEs are often not engaged or are dismissed because they are perceived as being less proficient in context‐specific terminology and in understanding roles, procedures and policies.^13^ The assumption that engagement requires cumbersome unidirectional capacity‐building whereby PWLE must be ‘brought up to speed’ is inherently flawed. The literature places great emphasis on preparing PWLE to engage in CPD while deflecting attention away from the need to also better prepare those working with and learning from PWLE.^12^, ^15^ By focusing solely on building capacity among PWLE, initiatives fail to make space for mutual learning or to challenge dominant beliefs about legitimate forms of knowledge, both of which are critical to meaningful inclusion.^16^

## 5 RECOMMENDATIONS

While evidence supporting the inclusion of lived experience in education mounts across health disciplines, there remains a significant lack of scholarly guidance on how to establish and engage in these partnerships. Drawing on the limited body of scholarship and on our learned and lived expertise, we offer initial thoughts to consider when striving to forge equitable partnerships with PWLE in CPD initiatives.

### 5.1 The process is as important as the outcome

Prioritizing the process of engagement, and investing time and resources from the outset, allows expectations and support needs to be identified for everyone involved (i.e., educators, programme leaders, PWLE, learners). A key factor in establishing meaningful educational partnerships is the ongoing commitment to shift from tokenistic modes of participation to more meaningful forms of engagement.^17^

### 5.2 Critical self‐reflection presents an opportunity to recognize diverse forms of knowledge

Working across different areas of expertise requires thinking differently about what counts as knowledge(s), including questions pertaining to the necessary and sufficient conditions for knowledge creation, scope of knowledge and limits of knowledge. The epistemological process of reflecting on values and beliefs—at the individual, programme and organizational levels—is essential to the success of working relationships. A critically reflexive perspective within CPD, and more broadly within healthcare programmes and service delivery, presents opportunities to bridge gaps between theory and practice.

Before engaging PWLE in CPD initiatives, it is useful to examine one's own values and beliefs in action. For example, reflect on your assumptions about engagement, preferences, experiences, expectations and boundaries.^18^ One approach to developing a feasible strategy begins by delineating the why, who, how and what of lived experience engagement in CPD initiatives. Examples of critically reflexive questions and sample actions are provided in Table 2.

**Table 2:** Sample critically reflexive questions and actions.

	Critically reflexive questions	Actions
Why?	Why are we engaging in this work and what are the key drivers of engagement?	Assess individual, programme and organizational readiness to engage.
	Why now?	Embed a commitment to engaging PWLE within organizational documents (e.g., mission statements).^8^
		Reflect on the timing of the initiative—is there sufficient lead time to build relationships with PWLE and to engage them from end‐to‐end?
Who?	Who is well suited for the role(s)?	Recruit two or more PWLE as part of a commitment to having multiple lived experience perspectives.
	How many people should be recruited?	
	Whose voices are included/excluded?	Create opportunities for training, capacity‐building and peer support^19^ for all team members.
	Who is well‐positioned to offer support and guidance to PWLE?	
	Who can be a champion for change within the organization?	
How?	How can we create supportive practices and processes?	Provide training and orientation for all team members.
	How will we engage in ongoing assessment?	Create opportunities for relationship‐ and trust‐building on the team.
	How will we evaluate our approach?	Co‐develop supportive policies and practices, including compensation for planning, participation and evaluation.^19^
	How will we foster supportive environments for collaboration?	Explore complementary skill sets and perspectives on the team and how to create space for all team members to share those perspectives.
	How will we navigate decision‐making, conflict and differences of perspective?	Set aside time for regular debrief discussions.^19^
	How will we hold ourselves accountable for our engagement strategy?	Establish channels for formal and informal feedback to improve the programme and increase the quality of the experience for PWLE.
What?	What role will PWLE have in the programme, educational initiative or team?	Support autonomy and self‐determination with respect to role title and language, and the level and scope of involvement.
	What have we done to clearly articulate and communicate the expectations of all team members?	Collaborate on curriculum development, didactic presentations, programme evaluations and so on.
	What biases or assumptions are shaping our perspectives and decisions?	Consider leadership and supportive roles for PWLE.
	What training and/or resources are needed to foster receptive contexts for lived experience engagement and leadership?	Explore openness to share and relinquish power.

### 5.3 Understand intentions and motivation for doing this work

Reflecting on the question ‘Why now?’ can help to surface organizations' motivation for change, as well as their readiness and commitment to engaging PWLE in CPD initiatives. Asking ‘Why now?’ also reflects an understanding that engagement does not occur in isolation, but rather is temporally situated and context‐driven.

### 5.4 Reflect on what knowledges are sought and for what purpose

Thoughtful consideration is necessary when deciding who to recruit, how many people to recruit, what knowledges and perspectives are being sought for the role and whose voices are included and excluded in the process. Offering intentionality when creating equitable partnerships and power‐sharing can avoid the tokenistic engagement of PWLE. Tokenism refers to the practice of seeming to involve PWLE in decision‐making when in fact their involvement is perfunctory.

### 5.5 Commit to sharing power

The collaborative approaches discussed in this paper require power‐sharing, as well as individual and organizational commitments to challenge existing power dynamics. This could include considering how decisions are made (e.g., hierarchical decision‐making, shared decision‐making, co‐production). There are also numerous opportunities to increase choice, autonomy and self‐determination for PWLE in CPD. We recommend that educators carefully consider leadership and supportive roles for PWLE in these programmes. Power is a central and crucial consideration, including the power‐equalizing conditions in place and the openness of leadership to share and relinquish power.^20^

## 6 CONCLUSION

Meaningful inclusion of PWLE in CPD initiatives for mental health professionals can bridge gaps between theory, practice, academia and community involvement, and can also reduce stigma and social distance between those with lived and learned expertise. To date, little guidance has been provided on how to meaningfully engage PWLE of engagement with the mental health system in CPD initiatives for mental health professionals. Beyond process and policy change, we feel that meaningful engagement requires a shift in perspectives at all levels. By engaging PWLE in CPD for mental health professionals through critical reflexivity, CPD initiatives can become more relevant, impactful, authentic, vibrant and consistent with a larger vision of systemic equity.

## AUTHOR CONTRIBUTIONS

**Holly Harris**: Conceptualization; project administration; writing—original draft; writing—review and editing. **Chantalle Clarkin**: Conceptualization; writing—original draft; writing—review and editing. **Jordana Rovet**: Conceptualization; writing—original draft; writing—review and editing. **Allison Crawford**: Conceptualization; writing—review and editing. **Andrew Johnson**: Conceptualization; writing—original draft; writing—review and editing. **Anne Kirvan**: Conceptualization; writing—original draft; writing—review and editing. **Sam Gruszecki**: Conceptualization; writing—original draft; writing—review and editing. **Stephanie Wang**: Conceptualization; writing—original draft; writing—review and editing. **Sophie Soklaridis**: Conceptualization; supervision; writing—original draft; writing—review and editing.

## CONFLICT OF INTEREST STATEMENT

The authors declare no conflict of interest.

## Data Availability

Data sharing is not applicable to this article as no data sets were generated or analysed during the current study.
